# Mechanical Properties and Damage Evolution of Concrete Materials Considering Sulfate Attack

**DOI:** 10.3390/ma14092343

**Published:** 2021-04-30

**Authors:** Qianyun Wu, Qinyong Ma, Xianwen Huang

**Affiliations:** 1 School of Civil Engineering and Architecture, Anhui University of Science and Technology, Huainan 232001, China; wqyahust@126.com; 2 Engineering Research Center of Underground Mine Construction, Ministry of Education, Anhui University of Science and Technology, Huainan 232001, China; 3 School of Civil and Architecture Engineering, Jiangsu University of Science and Technology, Zhenjiang 212003, China

**Keywords:** concrete, sulfate attack, physical and mechanical properties, damage model, microstructure

## Abstract

In order to study the durability of concrete materials subjected to sulfate attack, in a sulfate attack environment, a series of concrete tests considering different fly ash contents and erosion times were conducted. The mechanical properties and the micro-structure of concrete under sulfate attack were studied based on the following: uniaxial compressive strength test, split tensile test, ultrasonic impulse method, scanning electron microscopy (SEM) and X-ray diffraction (XRD). The mechanical properties were compressive strength, splitting tensile strength, and relative dynamic elastic modulus, respectively. Additionally, according to the damage mechanical theory, experimental results and micro-structure analysis, the damage evolution process of concrete under a sulfate attack environment were studied in detail. Finally, according to the sulfate attack time and fly ash content, a damage model of the sulfate attack of the binary surface was established. The specific results are as follows: under the action of sulfate attack, the change law of the rate of mass change, relative dynamic modulus of elasticity, corrosion resistance coefficient of compressive strength, and the corrosion resistance coefficient of the splitting tensile strength of concrete all increase first and then decrease. Under the same erosion time, concrete mixed with 10% fly ash content has the best sulfate resistance. Through data regression, the damage evolution equation of the sulfate attack was developed and there is an exponential function relationship among the different damage variables. The binary curved surface regression effect of the concrete damage and the erosion time and the amount of fly ash is significant, which can predict deterioration of concrete damage under sulfate attack. During the erosion time, the combined expansion of ettringite and gypsum caused micro cracks. With an increase of corrosion time, micro cracks developed and their numbers increased.

## 1. Introduction

Sulfate attack is an important factor affecting the service life and durability of concrete buildings and structures [1,2,3,4]. Soils containing a large amount of sulfate are common in saline areas of Northwest China, seawater in coastal areas, and groundwater. As a concrete structure is immersed in a sulfate solution for a long time, a large amount of sulfate ions from the solution is absorbed into the concrete, which reacts with hydration products to form ettringite precipitation. This gradually generates stress on the inner walls of pores, leading to deformation of and damage to the concrete structure [5,6,7]. This, severely affects the safety and service life of the concrete structure, and causes extensive economic losses [8,9]. Therefore, studying the damage degradation process of concrete under sulfate attack and the damage model of sulfate attack is helpful to delay erosion damage to concrete structures and evaluate their service states. At present, many scholars have carried out a great deal of research on the sulfate corrosion resistance of concrete and have achieved fruitful results [10,11,12,13,14]. The right amount of fiber can inhibit the generation and expansion of microcracks in concrete. This enhances mechanical properties [15] and improves the durability of concrete, and shows that the effect of mixed fiber is better than that of single fiber [16,17,18]. In addition, adding an appropriate amount of fly ash to replace cement in fiber concrete can improve its durability [19,20,21].

At present, scholars at home and abroad have primarily studied the change law and erosion mechanisms of the macroscopic mechanical properties of concrete under sulfate attack [22,23]. At the same time, most scholars have established damage models that take compressive strength [24], split tensile strength, or relative dynamic elastic modulus as a single evaluation index of the damage variable to measure the damage caused by sulfate erosion of concrete. However, little attention has been devoted to the relationship between the amount of damage of each evaluation index [25,26]. Moreover, a single factor model with the time of sulfate erosion as the main variable was also established. Bao et al. [27] conducted tensile tests on concrete after sulfate erosion and established an evolution model with crack number density as the damage degree; they found that this model could better reflect the evolution law of concrete erosion damage. An et al. [28] conducted sulfate erosion tests on recycled concrete and established a parabolic damage model with *E*_r_ as the damage quantity and found that it could better reflect the evolution law of erosion damage. Wu et al. [29] studied the relationship between ITZ (interface transition zone)and the damage evolution of concrete under the action of sodium sulfate erosion. They found that the influence of ITZ on the damage evolution of concrete was related to binder composition and immersion time. Xiao et al. [30] carried out a sulfate freeze–thaw coupling test on concrete and established a damage equation using the two-factor Weibull distribution model; they found that the damage fitted by the Weibull damage equation had a good correlation with RAC. Most scholars established concrete damage models that act on single factors; however, the performance of concrete subjected to sulfate erosion is the result of the combined actions of erosion time and the constituent materials [31], as such establishing a damage model based on both erosion time and the constituent materials should be considered.

In this paper, fly ash content and erosion time are taken as the main variables, while the uniaxial compressive strength test, split tensile test, ultrasonic testing test, scanning electron microscopy (SEM) and X-ray diffraction (XRD) were carried out on concrete under sulfate attack. First, the rate of mass change, relative dynamic modulus of elasticity, corrosion resistance coefficient of compressive strength, and the corrosion resistance coefficient of splitting tensile strength of concrete of different erosion times were analyzed. The erosion damage of concrete caused by sulfate attack was defined by the strength damage and wave velocity damage. Considering the superimposed effect of fly ash and erosion time, the influence of fly ash and sulfate erosion time on the expansion of concrete damage was discussed. Based on fly ash content and sulfate erosion time, a concrete composite erosion damage model was established. Finally, our study reveals the microstructure change law of concrete subjected to sulfate attack, aiming to provide theoretical support and an experimental basis for durability research of concrete structures in areas where sulfate attack occurs.

## 2. Materials and Methods

### 2.1. Raw Materials and Concrete Mixture Proportions

**Figure 1:**
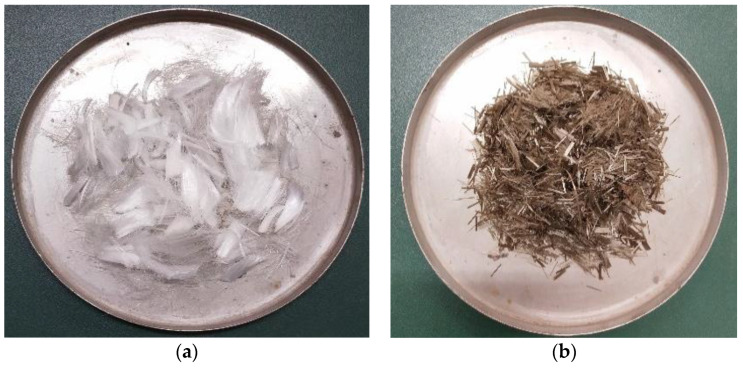
The appearance of fiber. (**a**) Polypropylene fiber and (**b**) basalt fiber.

This study used Portland cement (P•O 42.5) produced by Bagongshan Cement Plant (Huainan, China), which conforms with the standard for Common Portland cement [32]. The chemical composition of the cement are given in Table 1. The fly ash employed was a secondary class fly ash and was purchased from Luoyang Yizhou Plastic Technology Co., Ltd. (Luoyang, China), which conforms to the standard for fly ash used for cement and concrete [33]. The chemical composition of the fly ash are listed in Table 1. Fly ash was used as a partial replacement for cement and its mixing amounts were 0, 10, and 20% (*ψ*, mass fraction) of the total cementitious material, respectively. The coarse aggregate used in the test was continuous grading crushed rock of 5–25 mm. The fine aggregate was river sand with fineness modulus of 2.6. The water used was fresh, potable laboratory tap water. In the experiments, 6-mm basalt fiber with a volume ratio of 0.1% and 12-mm polypropylene fiber with a volume ratio of 0.2% were used. Their appearances are shown in Figure 1. The physical and mechanical properties are listed in Table 2. A sodium sulfate solution was prepared using chemical analytical reagents.

According to the JGJ 55-2011 specification for the mix proportion of ordinary concrete [34], concrete with a strength grade of C30 was designed. Each group of concrete needed to have 3 parallel samples, and there were 108 concrete samples in total. In addition, the water-to-binder ratio (W/B) was 0.6. The mix ratios of concrete specimens are shown in Table 3.

**Table 1:** Chemistry composition of binder (%) [35,36].

Composition	SiO_2_	Al_2_O_3_	CaO	Fe_2_O_3_	SO_3_	MgO	Na_2_O	K_2_O
Cement	19.6	6.5	66.3	3.5	2.5	0.7	0.6	0.3
Fly ash	45.40	33.51	3.15	5.28	0.45	0.06	2.62	3.88

**Table 2:** Physical and mechanical properties of fibers [37].

Fiber Types	Basalt Fiber	Polypropylene Fiber
Density/(g·cm^−3^)	2.65	0.91
Melting point/°C	1450–1500	160
Elongation to fracture/%	3.2	30–50
Tensile strength/MPa	3000–3300	350–480
Elastic modulus/GPa	90–110	2.4–3.2

**Table 3:** Mix proportions of concrete.

Materials	FA0	FA10	FA20
Cement (kg∙m^−3^)	350	315	280
Fly ash (kg∙m^−3^)	-	35	70
Water (kg∙m^−3^)	210	210	210
Fine aggregate (kg∙m^−3^)	644	644	644
Coarse aggregate (kg∙m^−3^)	1196	1196	1196
Sand ratio (%)	35	35	35
Basalt fiber (%)	0.1	0.1	0.1
Polypropylene fiber (%)	0.2	0.2	0.2
Water–binder ratio (W/B)	0.6	0.6	0.6

Notes: FA—fly ash; 0.0, 10, 20—fly ash content of 0.0%, 10%, 20%, respectively.

The types and dosages of concrete and fiber designed in this paper were all carried out on the basis of the research done in [35,37]. Basalt fibers are high elastic fibers and polypropylene fibers are low elastic fiber. The two kinds of fibers play different roles in the process of concrete stress. The correct amount of a fiber disperses evenly within a concrete, enhances the crack resistance effect and improves the interface characteristic of the concrete. It also inhibits the internal stress of concrete cracking after preliminary initiation and, further, reduces the brittleness of concrete, improves the effect of crack resistance, and improves the tensile strength of hybrid-fiber-reinforced concrete materials.

### 2.2. Specimen Preparation

**Figure 2:**
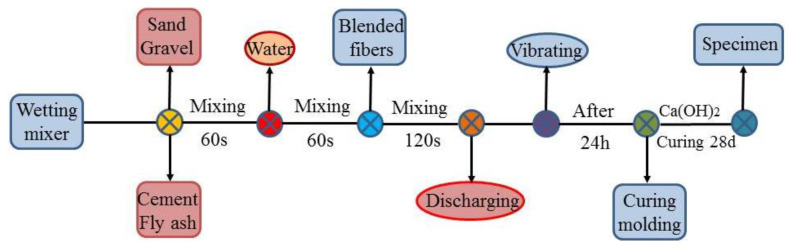
The mixing steps of concrete.

First, the sand, gravel, fly ash, and cement were batched and evenly mixed in a mixer for 60 s, and then the right proportion of water was added to the mixer for another 60 s. Second, blended fibers were evenly distributed into the solid mixture to prevent agglomeration which was mixed for 120 s. After mixing, the mixture was put into a cubic mold with side lengths of 100 mm × 100 mm × 100 mm, distributed evenly around the mold with a spatula to ensure compacting, and finally put on a vibrating table to seal all pores. The specimen was left for 24 h after loading into the mold, and then removed. The specimen was numbered and put into a saturated Ca(OH)_2_ solution with a relative humidity of 95% and a temperature of 20 ± 2 °C to cure for 28 days. The specific steps are shown in Figure 2.

### 2.3. Sulfate Attack Test for Concrete

After curing for 28 days under standard conditions, the concrete test block was placed in a sodium sulfate solution at a concentration of 5% for long-term immersion. The liquid level was 10 mm above the surface of the test block. In order to ensure the stability of the pH of the solution, the solution is replaced once a month, and the concrete was completely soaked and eroded. The times of sulfate attack were 0 d, 30 d, 60 d, 90 d, 120 d, and 150 d, respectively. The whole test was carried out at 25 ± 2 °C.

### 2.4. Other Tests

#### 2.4.1. Mass Change

When a specified erosion time was reached, concrete samples were taken from the solution and then dried in a constant temperature room at 22 °C until the mass was constant. At the same time, the sample surface was gently wiped with a towel to remove crumbs. The sample mass was then weighed and recorded using an electronic scale with an accuracy of 0.01 g. There were 3 parallel concrete samples in each group when weighing, and the results were averaged. Next, to calculate the rate of mass change Equation (1) was used [38]:

(1) $$\varphi_{i}=\left(m_{n}-m_{0}\right)/m_{0}$$

where $\varphi_{i}$, $m_{n}$, $m_{0}$ are the rate of mass change of the concrete specimen, the mass of the specimen after erosion time *n*, and the initial mass (g), respectively.

#### 2.4.2. Ultrasonic Impulse Method

**Figure 3:**
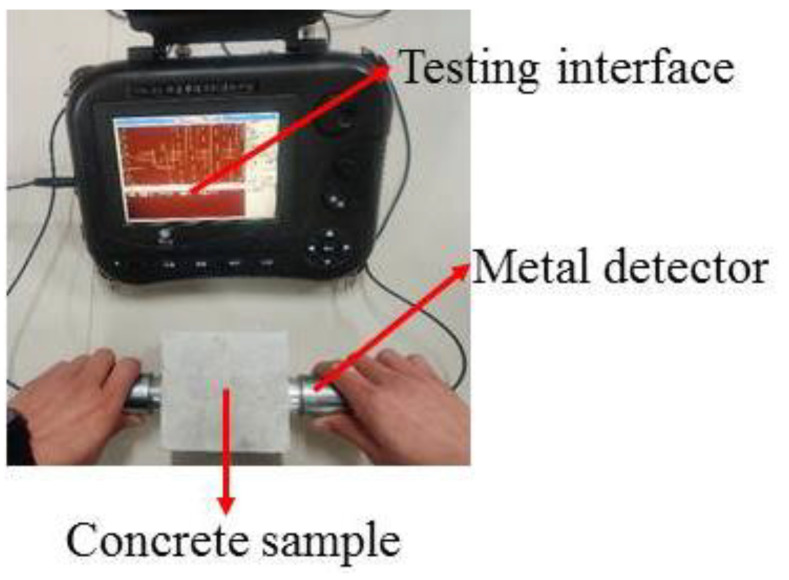
NA-M4 nonmetal ultrasonic detector.

**Figure 4:**
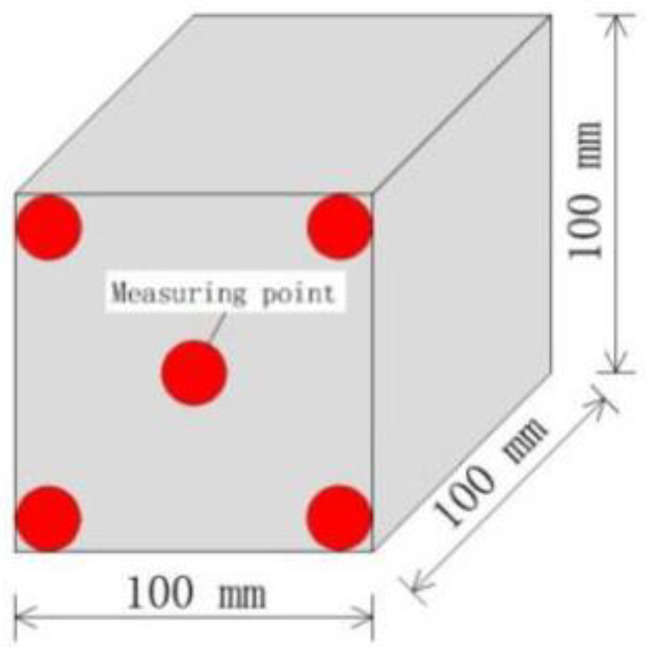
Measuring point positions of the detector.

After the specified number of sulfate attack, each test piece was removed. An NA-M4 nonmetal ultrasonic detector (shown in Figure 3) was used to measure the ultrasound velocity in the 100-mm cube of concrete. The transducers that were used were 50 mm thick. The ultrasonic method was used to measure the longitudinal wave velocity passing through the concrete samples. The path length between the sensors was 100 mm, the length of the dimensions of a concrete sample. Five pairs of measurement points (shown in Figure 4) were used to measure the ultrasound wave velocity and the average was taken. To ensure proper coupling of the transducer to the surface being measured, Vaseline was evenly applied to the transducer probe. For the entire test process, the ultrasonic frequency was 50 kHz, the transmission voltage was set to 500 V, and the sampling period was 0.4 μs.

The longitudinal wave velocity was calculated using Equations (2) and (3).

(2) v=l/t

(3) $$t=\left(t_{1}+t_{2}+t_{3}+t_{4}+t_{5}\right)/5$$

where *v* is the longitudinal wave velocity (m/s); *l* is the path length (mm); *t* is the average ultrasound time (μs); *t*_1_, *t*_2_, *t*_3_, *t*_4_, and *t*_5_ are the ultrasound time values of 5 pairs of measuring points, respectively.

According to the relationship between the dynamic elastic modulus of the material and the ultrasonic sound velocity, the relative dynamic elastic modulus *E_r_*(*n*) according to the GB/T 50082-2009 standard for both test methods, for long-term performance and durability of ordinary concrete [36], can be expressed as Equation (4).

(4) $$E_{r}(n)=E_{n}/E_{0}=v_{n}^{2}/v_{0}^{2}$$

where $E_{r}(n)$, $E_{n}$, $E_{0}$ are the relative dynamic elastic modulus of the concrete specimen, the dynamic elastic modulus after erosion time *n*, and the initial dynamic elastic modulus, respectively; $v_{n}$, $v_{0}$ are the longitudinal wave velocity (m/s) of the concrete specimen after erosion time *n* and the initial longitudinal wave velocity (m/s).

#### 2.4.3. Mechanical Test

**Figure 5:**
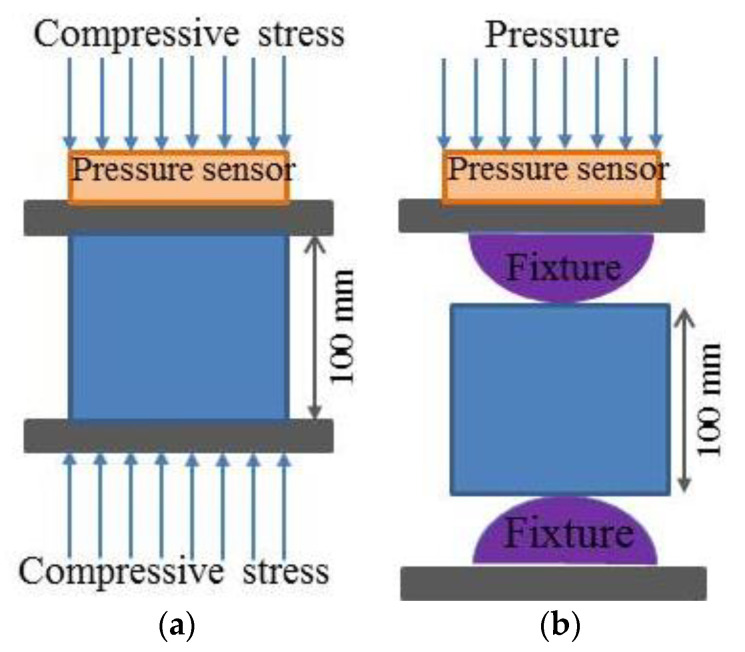
Static compression and splitting tensile tests of specimens. (**a**) Static compression test and (**b**) splitting tensile test.

When each set of sulfate erosion times was reached, the concrete sample was taken out of the solution and dried at room temperature. According to the GB/T 50081-2019 standard for test methods for the physical and mechanical properties of concrete [39], a CSS-YAN3000 press, produced by the Changchun Testing Machine Institute (Changchun, China), was used for uniaxial compressive strength tests of concrete (shown in Figure 5a) as well as split tensile tests (shown in Figure 5b); the loading rates were 3 mm/min and 1 mm/min, respectively. There were 3 parallel concrete samples in each group during the strength tests, and the results are averaged. The loading mode is shown in Figure 5. The corrosion resistance of concrete was reflected by the corrosion coefficient indexes of the compressive and tensile strengths of concrete, before and after being eroded by the sulfate solution. The calculation formulas are shown below (Equations (5) and (6)).

(5) $$k_{c}=f_{cn}/f_{c0}$$

(6) $$k_{t}=f_{tn}/f_{t0}$$

where $k_{c}$, $f_{cn}$, $f_{c0}$ are the corrosion resistance coefficient of the compressive strength of a concrete specimen, the compressive strength (MPa) after erosion time *n*, and the uncorroded compressive strength (MPa), respectively; $k_{t}$, $f_{tn}$, $f_{t0}$ are the corrosion resistance coefficient of the splitting tensile strength of a concrete specimen, the split tensile strength (MPa) after erosion time *n* and the split tensile strength (MPa) without sulfate attack, respectively.

#### 2.4.4. Microanalysis

SEM and XRD tests were carried out using an S-3400N scanning electron microscope and a SMARTLAB9KW X-ray diffractometer from Yunnan Langlue Technology Co., Ltd. The microstructures, morphologies, and erosion products of minerals in the eroded samples were tested. The purpose was to analyze the erosion damage mechanism from a microscopic point of view. When a specified time of sulfate attack was reached, the concrete sample was taken out of the solution, left to air dry at room temperature, and broken into smaller parts for testing. After being placed in an oven and dried at 40 °C for 24 h, some samples were taken out, their surfaces were plated with gold on the surface, and then subjected to SEM tests. At the same time, the remaining test samples were crushed into powder and analyzed using XRD.

## 3. Experimental Results and Discussion

### 3.1. Characteristics of Apparent Degradation

**Figure 6:**
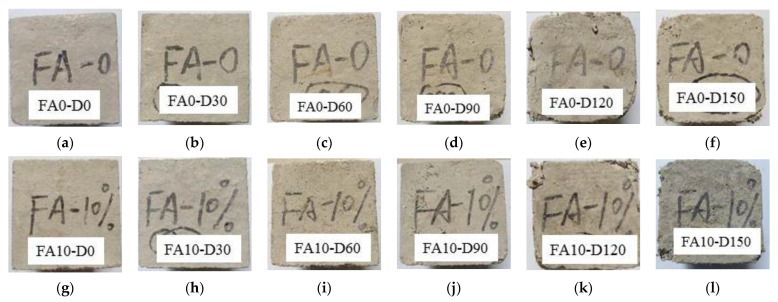

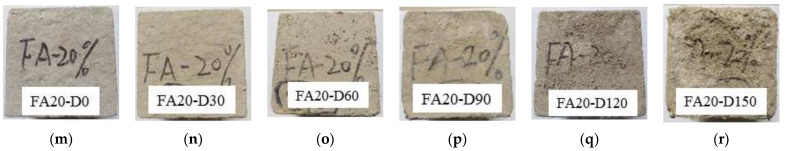
Deformation appearances of concrete samples subjected to sulfate attack. (**a**) FA0-D0; (**b**) FA0-D30; (**c**) FA0-D60; (**d**) FA0-D90; (**e**) FA0-D120; (**f**) FA0-D150; (**g**) FA10-D0; (**h**) FA10-D30; (**i**) FA10-D60; (**j**) FA10-D90; (**k**) FA10-D120; (**l**) FA10-D150; (**m**) FA20-D0; (**n**) FA20-D30; (**o**) FA20-D60; (**p**) FA20-D90; (**q**) FA20-D120; and (**r**) FA20-D150.

The appearance and damage patterns of the concrete samples with different fly ash contents that were completely immersed in 5% sodium sulfate solution for different times are shown in Figure 6.

The surface of the concrete specimens changed during erosion in the sodium sulfate solution with the increase in erosion time. The surface of various concretes eroded by 0-d sulfate were relatively flat and smooth. After 30 d of sulfate attack, minor defects began to appear on various concrete surfaces. After 60 d of sulfate attack, the surfaces of various concrete samples went from being relatively smooth surfaces to rough surfaces, and the mortar gradually peeled off. After 120 d of sulfate attack, the corners of the concrete of the FA0 group and FA10 group fell off dropped slightly and the surface mortar peeled off, while the surface mortar of the concrete specimens from the FA20 group appeared to have mostly peeled off. After 150 d of sulfate attack, cracks occurred at the corners and ends of the concrete in the FA0 and FA10 groups and the mortar peeled off; the corners of the concrete specimens in the FA20 group developed cracks. The more mortar completely peeled off of the surface of the entire concrete specimen, the more severe the deterioration. The reason for this is that the structure of a concrete test block was seriously damaged by sulfate erosion and the surface became brittle. A large amount of sulfate gathered in the concrete, and sulfate crystals precipitated after water evaporation [40,41].

### 3.2. Rate of Mass Change, Relative Dynamic Modulus of Elasticity and Corrosion Resistance Coefficient of Strength

**Figure 7:**
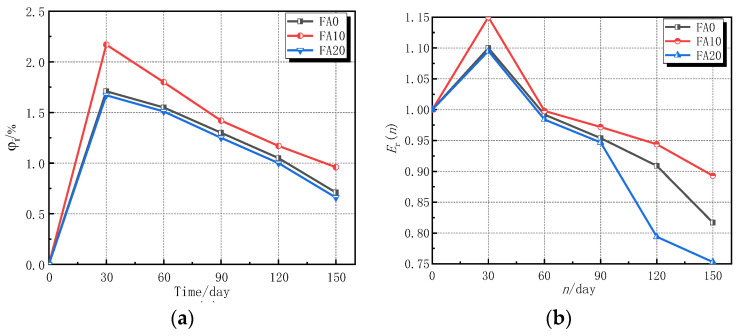

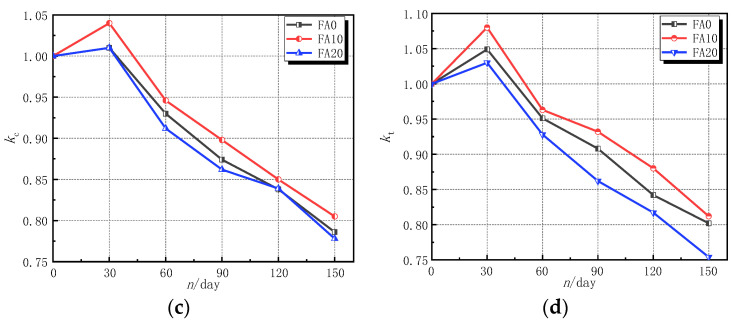
Testing results. (**a**) Variation of mass; (**b**) variation of *E*_r_(*n*); (**c**) variation of *k*_c_; and (**d**) variation of *k*_t_.

According to Formulae (1) and (4)–(6), the relationships among the rate of mass change, relative dynamic elastic modulus, and corrosion resistance coefficient of strength of concrete specimens under the action of sulfate erosion and erosion time were obtained, as shown in Figure 7.

The change law of the rate of mass change, relative dynamic elastic modulus, and corrosion resistance coefficient of strength of different erosion times of sulfate attack are shown in Figure 7. It can be seen from Figure 7 that with an increase of erosion time, the rate of mass change, relative dynamic elastic modulus, and corrosion resistance coefficient of strength of concrete specimens with different fly ash contents all initially increase and then decrease. When the fly ash content was 10%, concrete had the best resistance to sulfate attack [42]. The reason for this is that the active substances in fly ash reacted with the Ca(OH)_2_ in concrete, which reduced the content of Ca(OH)_2_ in the concrete, reduced the production of gypsum and ettringite, and also alleviated the expansion of crystals. At the same time, because of the micro-aggregate effect of fly ash, fly ash particles filled the space between the unhydrated cement particles, which reduced the internal porosity of concrete, improving the pore structure and compacting the internal structure of concrete; this improved the durability of concrete [43]. At 30 d of sulfate attack, the mass of concrete, relative dynamic elastic modulus, corrosion resistance coefficient of compressive strength, and corrosion resistance coefficient of the split tensile strength with 10% fly ash increased by 2.17%, 10%, 1.0%, and 3.0%, respectively, compared to uncorroded concrete. The mass of concrete, relative dynamic elastic modulus, and corrosion resistance coefficient of strength decreased gradually after 60 d of erosion. The mass of concrete, the relative dynamic elastic modulus, and corrosion resistant coefficient of strength increased with the extension of the first corrosion time after decreasing initially. The reason being that early sulfate, ettringite, gypsum, and other products are produced by the reaction of sodium sulfate solution with hydration products of concrete. At the same time, sulfate intrudes into the concrete specimen, which fills and compacts the pores and cracks within the concrete. The concrete specimen is more compacted than before the erosion and the mass of concrete, relative dynamic elastic modulus, and corrosion resistance coefficient of strength of the concrete are increased. With the continuation of sulfate erosion and the continuous accumulation and expansion of products, micropores and microcracks inside the concrete expand and extend. At the same time, the concrete surface mortar peels off, and the mass of concrete, relative dynamic elastic modulus, and corrosion resistance coefficient of strength gradually begin to decrease.

## 4. Establishment of Evolution Model of Sulfate Erosion Damage

### 4.1. Model of Erosion Damage Based on Each Evaluation Index

It can be seen from the sulfate attack test that the mechanical properties of concrete changed with the increase in erosion time, and the changes in the macrophysical properties can reflect the degree of internal changes in concrete materials. In order to quantitatively reflect the change law of the mechanical properties of concrete under the action of sulfate erosion, and comprehensively evaluate the state of concrete change, using damage mechanics, the corrosion resistance coefficient of compressive strength (*k_c_*), the corrosion resistance coefficient of splitting tensile strength (*k_t_*), and longitudinal wave velocity (*v*) were selected as damage variables. The damage caused by sulfate attack is shown by Equations (7)–(9).

(7) $$D_{1}=1-k_{c}$$

(8) $$D_{2}=1-k_{t}$$

(9) $$D_{3}=1-v_{n}/v_{0}$$

where *D*_1_, *D*_2_, *D*_3_ are the sulfate erosion damage variables and are the corrosion resistance coefficient of compressive strength, the corrosion resistance coefficient of splitting tensile strength, and the longitudinal wave velocity, respectively.

**Figure 8:**
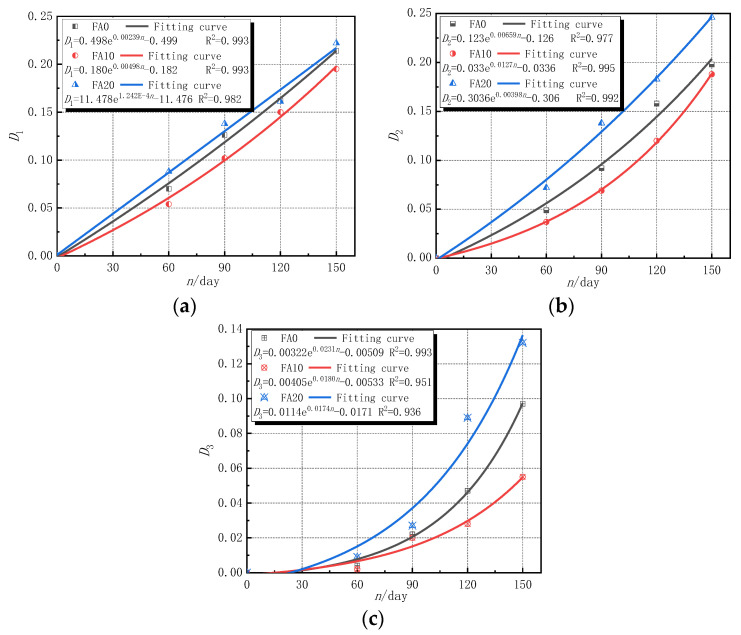
Relationship between damage variable of different evaluation indexes and erosion times. (**a**) Results of *D*_1_; (**b**) results of *D*_2_; and (**c**) results of *D*_3_.

When the times of sulfate attack of concrete are 0 to 30 d, the sodium sulfate solution reacted with hydration products in concrete to produce ettringite, gypsum, and other products, which filled the initial holes in concrete, causing the concrete to be more compact. However, when the erosion time exceeded 30 d, that is, after 60 d of sulfate attack in this experiment, as the erosion time increased, the erosion intensified, and the damage evolution process of concrete changed with length of time. According to Equations (5)–(7), the amount of erosion damage under each evaluation index was calculated, as shown in Figure 8. The established equation in Figure 8 is only valid for concrete composed of polypropylene fiber, basalt fiber, and fly ash subjected to sulfate attack for 60–150 days.

It can be seen from Figure 8 that, with the increase in the time of sulfate attacks, the erosion damage defined by the three indexes of the corrosion resistance coefficient of compressive strength, the corrosion resistance coefficient of splitting tensile strength, and the wave velocity increased continuously. The erosion damage and damage degradation rate of concrete specimens with 10% fly ash content were significantly lower than those without fly ash content. The reason for this was that an appropriate amount of fly ash can fully react with the internal components of concrete to generate a C-S-H gel and other substances, enhance the internal cohesion of concrete, reduce the porosity of concrete, improve its compactness, improve its ability to resist sulfuric acid erosion, and improve the ability to resist spallation [44]. However, when the fly ash content was 20%, the erosion damage and damage degradation rate of concrete specimens were higher than those without fly ash content. The reason for the erosion damage to ash was that an appropriate amount of fly ash can consume a certain amount of Ca(OH)_2_, which reduces the content of substances that react with SO_4_^2−^, thereby reducing the generation of erosion products [35,37]. Under the same sulfate attack, the damage defined by the wave velocity index was less than the damage defined by the strength corrosion resistance coefficient index. The reason being that the damage of concrete from sulfate erosion is gradually weakened from the outside to the inside, and the longitudinal wave propagation velocity changed insignificantly, so the erosion damage defined by the wave velocity was relatively small [11,45].

At the same time, it can also be seen from Figure 8 that the correlation coefficients of the fitting formulas of the sulfate erosion damage variables are relatively high, which can better fit the damage evolution law of concrete specimens over time under the action of sulfate erosion. After data fitting, the erosion damage evolution of each performance index showed a more obvious exponential function relationship, and the general fitting function formula is shown in Formula (10).

(10) $$D_{n}=ae^{bn}+c$$

where *n* is the erosion time; *a*, *b*, and *c* are the coefficients in the fitting formula, as shown in Figure 8a–c.

**Figure 9:**
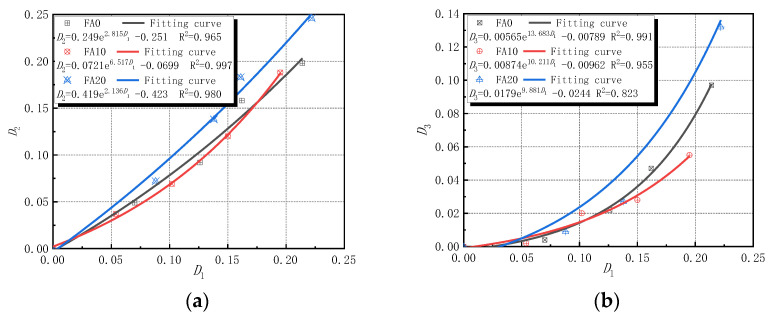
Relationship between different evaluation indexes of sulfate erosion damage. (**a**) Relationship between *D*_2_ and *D*_1_ and (**b**) relationship between *D*_3_ and *D*_1_.

The relationship between the sulfate erosion variables *D*_2_ and *D*_1_ is established, as shown in Equation (11).

(11) $$D_{2}=a_{1}e^{b_{1}D_{1}}+c_{1}$$

where *a*_1_, *b*_1_, and *c*_1_ are the coefficients in the fitting formula, as shown in Figure 9a.

There is also a good exponential relationship between damage amount *D*_1_ of the corrosion resistance coefficient of compressive strength and ultrasonic velocity damage amount *D*_3_ of the non-destructive testing, as shown in Figure 9b. Thus, the longitudinal wave velocity of the non-destructive testing can be used to predict the strength performance of and damage to the concrete structure.

### 4.2. Erosion Damage Model of Corrosion Resistance Coefficient of Compressive Strength Based on the Erosion Time and Fly Ash Content

**Figure 10:**
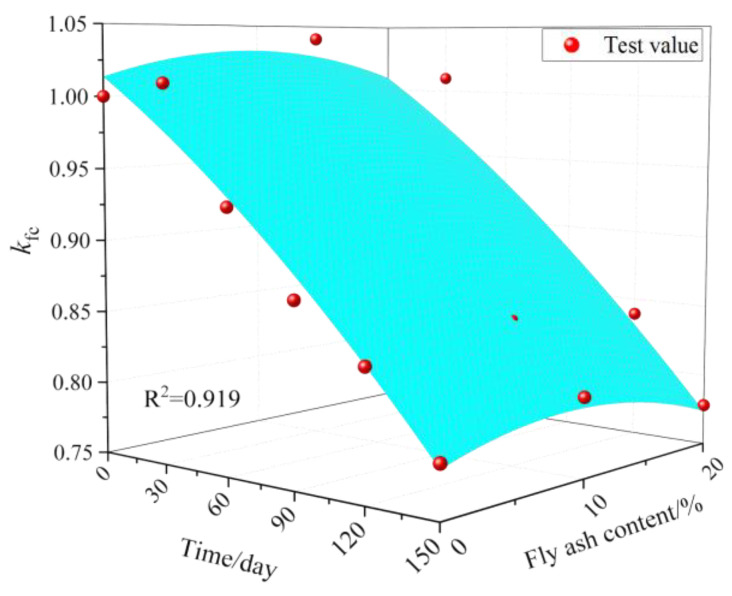
Relationship between fly ash content and erosion time *n* with *k*_c_.

To better study the influence of sulfate attack times and fly ash content on the corrosion coefficient of concrete’s compressive force, a sulfate damage prediction model using both sulfate attack time factors and fly ash content was examined. The scatter diagram results of sulfate erosion times, the amount of fly ash, and the corrosion resistance coefficient of compressive strength of the concrete specimens are shown in Figure 10. The mathematical model of sulfate damage established based on regression analysis is shown in Formula (12). The correlation coefficient of data regression analysis was 0.919, the fitting coefficient was relatively high, and the fitting surface can be in good agreement with the experimental value. This shows that this model can be used to predict the quantitative relation between concrete erosion damage and the amount of sulfate attack and the amount of fly ash after sulfate attack, so as to evaluate the corrosion resistance of concrete under sulfate attack. The model is only established based on the experimental data; hence, the model is not universal. It can, however, provide some calculation methods and data references for actual projects.

(12) $$k_{c}=1.014-0.00112n+0.00379\psi -2.844*10^{-6}n^{2}-1.992*10^{-4}\psi^{2}-1.476*10^{-6}n\psi \;(R^{2}=0.919)$$

It can be seen from Table 4 that the error between the calculated values and the measured values of the regression formula is small. Among these, the minimum error without fly ash occurred at 120 d of sulfate erosion, with an error of 0.12%. The maximum error was −3.17% at 30 d of sulfate attack. When the fly ash content was 10%, the minimum error occurred at 120 d of sulfate attack, with an error of 0.59%. The maximum error was −4.23% at 30 d of sulfate attack. When the fly ash content was 20%, the minimum error occurred with a sulfate erosion lasting 30 d, with an error of 0.30%. The maximum error occurred at 150 d of sulfate attack with an error of 4.49%. For engineering practices and measurement errors, this is negligible; hence, the test value and the fitting value are basically the same.

## 5. Mechanism Analysis

When concrete is subjected to sulfate attack, the change in its performance is, not only reflected in the process of macroscopic properties, but also in the process of microstructure changes.

### 5.1. XRD Analysis

**Figure 11:**
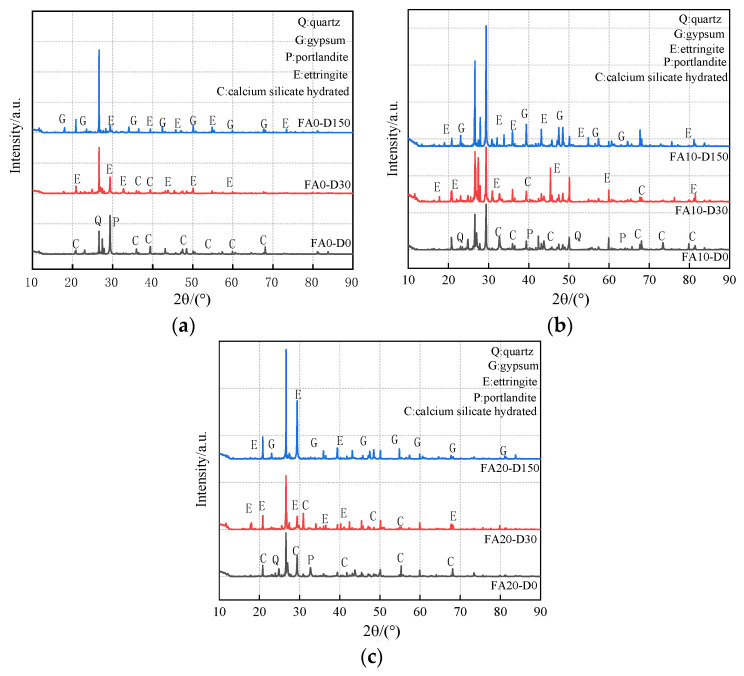
X-ray diffraction patterns of concrete subjected to sulfate attack. (**a**) FA0 concrete; (**b**) FA10 concrete; and (**c**) FA20 concrete.

Figure 11 shows the XRD mineral analysis results of various concretes with different erosion time stages (erosion times of 0 d, 30 d, 150 d). The results show that, when various concretes are not attacked by sulfate, the main components are quartz (SiO_2_), calcium hydroxide (Ca(OH)_2_), and calcium silicate hydrate (C-S-H). In addition to C-S-H, ettringite (Aft) could also be observed in groups FA0, FA10, and FA20 after being eroded for 30 days. At that time, ettringite production was limited, which filled the micro pores and cracks in the concrete specimens, optimizing the pore structure and making the concrete structure more compact. With the increase in erosion time, ettringite, and gypsum generation, when the erosion age reached 150 d, the content of Ca(OH)_2_ in the pores of concrete decreased continuously, decreasing the alkalinity and causing the erosion degree to intensify. A large amount of gypsum and ettringite was generated in the concrete, which increased in volume and produced expansion stress. When expansion stress exceeds the tensile stress of internal concrete, microcracks will form and the internal pore structure of concrete will be destroyed [11,46]. At the same time, it can be concluded that the amount of gypsum produced in concrete with fly ash is obviously more than that without fly ash, and the amount of gypsum produced increases with the increase in fly ash content [43]. More ettringite and gypsum were produced in specimens with fly ash at 150 days [43]. The product of fly ash concrete under sulfate erosion was similar to the experimental conclusions of Liu et al. [43] and Zhao et al. [11].

### 5.2. SEM Analysis

**Figure 12:**
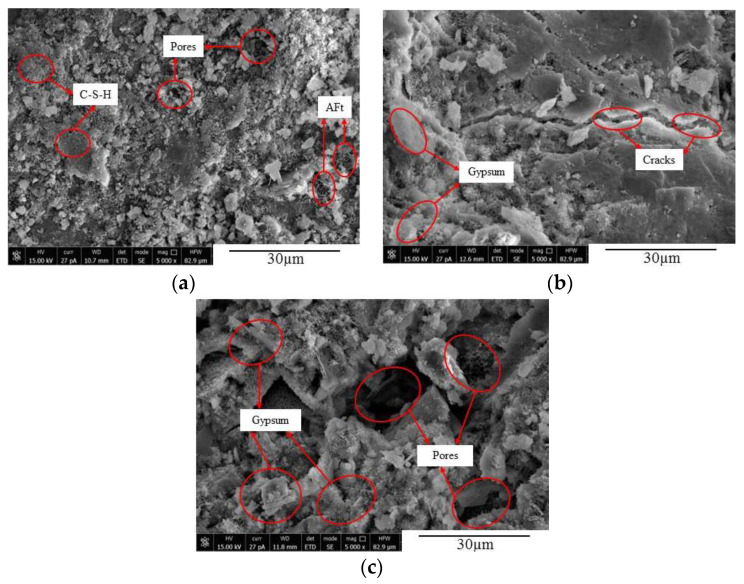
Microcrack expansion of FA0 concrete at different corrosion times. (**a**) FA0-D0; (**b**) FA0-D90; and (**c**) FA0-D150.

**Figure 13:**
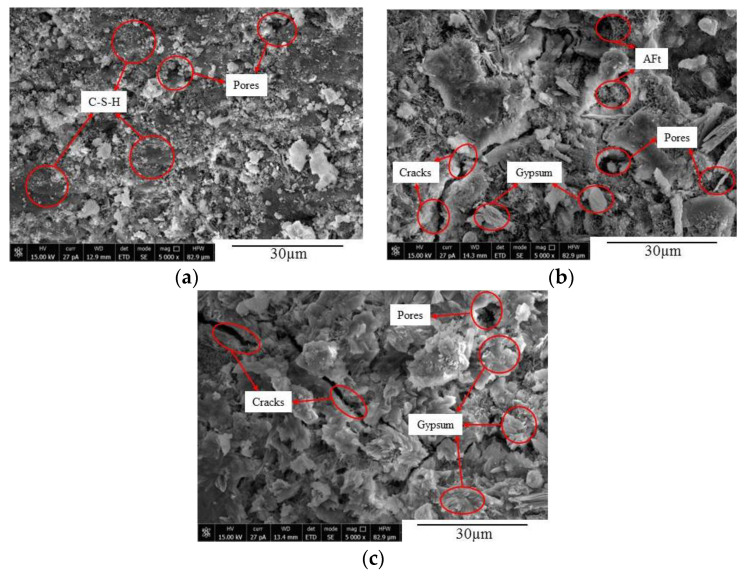
Microcrack expansion of FA10 concrete at different corrosion times. (**a**) FA10-D0; (**b**) FA10-D90; and (**c**) FA10-D150.

**Figure 14:**
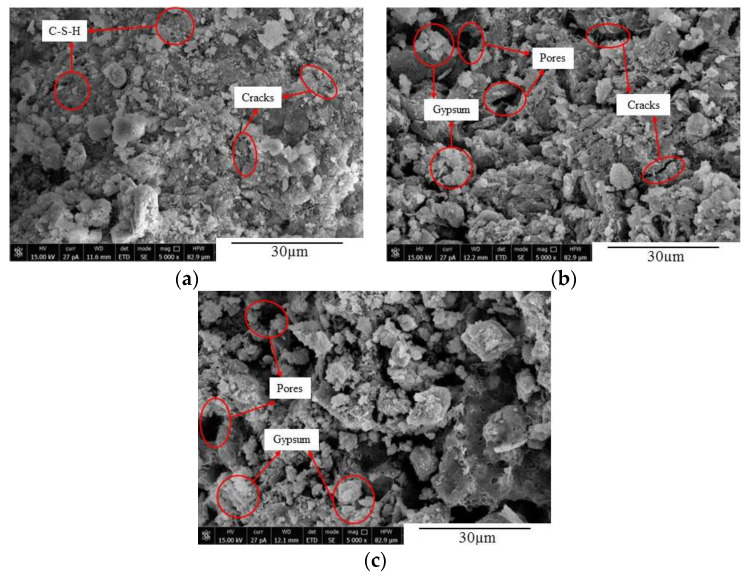
Microcrack expansion of FA20 concrete at different corrosion times. (**a**) FA20-D0; (**b**) FA20-D90; and (**c**) FA20-D150.

The microstructures of concrete at different time under the action of sulfate attack were observed using SEM, as illustrated in Figure 12, Figure 13 and Figure 14. It can be seen from Figure 12 that, when the FA0 group concrete was not corroded, there were numerous hydration products and C-S-H gel inside the concrete, with smaller pores and a denser internal structure (Figure 12a). After 90 d of erosion, a small amount of acicular calcium silicate, flaky gypsum, and cracks appeared inside the concrete, making the structure relatively loose (Figure 12b). After 150 d of sulfate attack, the erosion intensified, and more gypsum and ettringite formed in the concrete. During this process, volume expansion occurred, the expansion force increased, and the concrete cracked and expanded gradually (Figure 12c) [11,41]. Figure 13 shows that there were a large number of hydration products and C-S-H gel in various concretes before sulfate attack, and the internal structures were relatively complete and compact (Figure 13a). After 90 d of sulfate attack, a few new cracks appeared in the concrete from the FA10 group (Figure 13b). After 150 d of sulfate attack, more erosion products were generated, cracks increased and expanded, and the structure was loose. It can also be seen from Figure 14 that a small number of cracks appeared in the internal structure of concrete without sulfate attack. After 90 d of sulfate attack, a large number of new microcracks were generated in the internal structure of the FA20 group concrete, and the cracks crossed each other and the structure was loose (Figure 14b). As the erosion time increased, the microcracks in the concrete specimens expanded and increased, and the internal structure became loose, making the degree of erosion worse. After 150 d of erosion, the internal microstructure of FA20 concrete specimens further loosened, the cracks were interconnected, the internal space increased, and the compactness decreased (Figure 14c).

**Figure 15:**
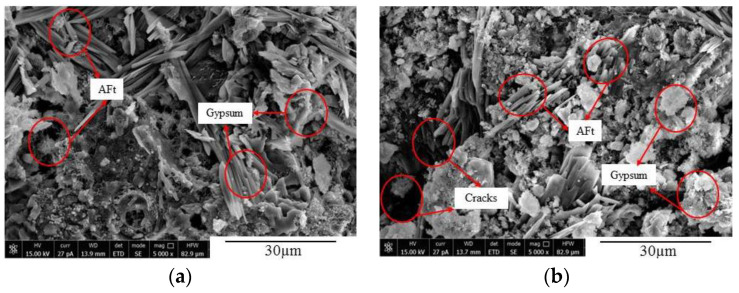
Growth and aggregation of products in FA10 concrete. (**a**) FA10-D90 and (**b**) FA10-D150.

As can be seen from Figure 15 that SO_4_^2−^ entered the concrete and reacted with its internal hydration products to form acicular ettringite crystals and flake-like gypsum, which was continuously generated in the microcracks and pores, thus making the pores inside the concrete compact. However, with the continuous diffusion and reaction of SO_4_^2−^, the generated needle-shaped ettringite and flaky gypsum gradually increased and crossed each other to form a network, which caused the pores of the concrete to be subjected to expansion forces. When the expansion forces reached a certain level, the number of microcracks and pores in the concrete specimens increased and expanded continuously [11]. According to the micromorphology of concrete after sulfate erosion, Zhang et al. [47] and Li et al. [9] observed that sulfate ions diffused into concrete and reacted with its internal substances to generate acicular ettringite crystals. With an increase in sulfate erosion time, in concrete, the amount of generated ettringite crystals gradually increased which finally led to the formation of cracks. The obtained microstructures and conclusions in this article are consistent with the results of Zhang et al. [47] and Li et al. [9].

## 6. Conclusions

According to the results of this paper, the following conclusions can be drawn:

(1) With an increase in sulfate attack time, the change law of concrete mass, relative dynamic elastic modulus, and strength corrosion resistance coefficient are similar, as they all initially increase and then decrease. Compared with other fly ash contents, concrete durability is better when fly ash content is 10%. The reason for this is that fly ash can fully react with the internal composition of concrete to produce C-S-H gel and other substances, enhance the internal cohesion of concrete, reduce the porosity of concrete, improve the density, and improve sulfuric acid erosion resistance.

(2) The corrosion resistance coefficient of compressive strength, the corrosion resistance coefficient of splitting tensile strength, and the change in wave velocity were taken as damage variables. Concrete sulfate erosion damage was comprehensively evaluated using various damage variables. The evolution equation of concrete sulfate erosion damage based on each damage variable was obtained by data regression, and the exponential function relationship between different damage variables was established.

(3) A composite sulfate erosion damage model between the corrosion resistance coefficient of compressive strength as a damage variable, erosion phase, and fly ash content was established. This model was used to predict concrete damage and erosion time after sulfate erosion. The model was used to predict the quantitative relationship between concrete damage, erosion time, and fly ash content after sulfate erosion to evaluate the resistance of concrete to sulfate attack in saline areas. The damage model is only suitable for concrete with a water–binder ratio of 0.6, fly ash dosage range of 0–20%, and mixed with 0.1% basalt fiber and 0.2% polypropylene fiber simultaneously.

(4) Sulfate attack changes the internal microstructure of concrete, and sulfate ions react with hydration products in concrete to produce expansive crystal ettringite and gypsum. With an increase in erosion products and expansion force, microcracks and damage appear inside the concrete, which is aggravated continuously, inducing the expansion and penetration of cracks and pores, and the deterioration of the microstructure reduces the macro performance of concrete.

## Figures and Tables

**Table 4:** Comparison of fitting *k*_*c*1_ and measured *k_c_*.

Time/Day	Fly Ash/%	Measured Data *k_c_*	Fitting Data *k*_*c*1_	Error$\frac{k_{c1}-k_{c}}{k_{c}}$/%
0	0	1	1.014	1.4
30	0	1.01	0.978	−3.17
60	0	0.93	0.937	0.75
90	0	0.874	0.890	1.83
120	0	0.838	0.839	0.12
150	0	0.786	0.782	−0.51
0	10	1	1.032	3.2
30	10	1.04	0.996	−4.23
60	10	0.946	0.954	0.85
90	10	0.898	0.907	1.0
120	10	0.85	0.855	0.59
150	10	0.805	0.798	−0.87
0	20	1	1.04	4.0
30	20	1.01	1.013	0.30
60	20	0.912	0.923	1.21
90	20	0.862	0.858	−0.46
120	20	0.839	0.871	3.81
150	20	0.778	0.813	4.49

## Data Availability

The data presented in this study are available on request from the corresponding author.

## Nomenclature

*n*	the erosion time
$\varphi_{i}$	the rate of mass change of the concrete specimen
$m_{n}$	the mass of the specimen after the erosion time *n*
$m_{0}$	the initial mass
*v*	the longitudinal wave velocity
*l*	the path length
t	the average ultrasound time
$t_{1}$	the ultrasonic time value of each pair of measuring points
$t_{2}$	the ultrasonic time value of each pair of measuring points
$t_{3}$	the ultrasonic time value of each pair of measuring points
$t_{4}$	the ultrasonic time value of each pair of measuring points
$t_{5}$	the ultrasonic time value of each pair of measuring points
$E_{r}(n)$	the relative dynamic elastic modulus
$E_{n}$	the dynamic elastic modulus after the erosion time *n*
$E_{0}$	the initial dynamic elastic modulus
$v_{n}$	the longitudinal wave velocity of the concrete specimen after the erosion time *n*
$v_{0}$	the initial longitudinal wave velocity
$k_{c}$	the corrosion resistance coefficient of compressive strength of the concrete specimen
$f_{cn}$	the compressive strength after the erosion time *n*
$f_{c0}$	the uncorroded compressive strength
$k_{t}$	the corrosion resistance coefficient of splitting tensile strength of the concrete specimen
$f_{tn}$	the split tensile strength after the erosion time *n*
$f_{t0}$	the split tensile strength without sulfate attack
*D*_1_	the sulfate erosion damage variables corresponding to the corrosion resistance coefficient of compressive strength
*D*_2_	the sulfate erosion damage variables corresponding to the corrosion resistance coefficient of splitting tensile strength
*D*_3_	the sulfate erosion damage variables corresponding to the longitudinal wave velocity
ψ	fly ash content
